# Perspective on the use of optics in bladder cancer detection and diagnosis

**DOI:** 10.1117/1.JBO.30.4.040601

**Published:** 2025-04-04

**Authors:** Marinka J. Remmelink, Dylan J. Peterson, Jakko A. Nieuwenhuijzen, Ton G. van Leeuwen, Joseph C. Liao, Daniel M. de Bruin

**Affiliations:** a Amsterdam University Medical Center Location University of Amsterdam, Department of Urology, Amsterdam, The Netherlands; b Cancer Center Amsterdam, Treatment and Quality of Life, Amsterdam, The Netherlands; c Stanford University School of Medicine, Department of Urology, Palo Alto, California, United States; d Amsterdam University Medical Center Location Vrije Universiteit, Department of Urology, Amsterdam, The Netherlands; e Amsterdam UMC Location University of Amsterdam, Department of Biomedical Engineering and Physics, Amsterdam, The Netherlands; f Cancer Center Amsterdam, Imaging and Biomarkers, Amsterdam, The Netherlands; g Veterans Affairs Palo Alto Health Care System, Palo Alto, California, United States

**Keywords:** optics, bladder cancer, diagnosis

## Abstract

**Significance:**

Bladder cancer (BC) diagnosis, management, and outcomes depend on the accurate detection of tumors via optical technologies. Accordingly, understanding the benefits and limitations of these technologies permits improvements in patient care and identifies areas for future research.

**Aim:**

We outline the current process of BC detection and diagnosis, explore the current role of optical technologies, and discuss the opportunities and challenges they present in this field.

**Approach:**

The current diagnostic pathway of BC, the use of optical technologies, and their shortcomings in this process are reviewed. From there, opportunities and challenges of optics in BC detection and diagnosis are discussed.

**Results:**

BC management is expensive due to the limitations of white light cystoscopy, the requirement for histopathological confirmation, and the need for long-term surveillance. Alternative non-optical methods lack accuracy, and available optical techniques focus only on cancer detection. Alternatives to histopathology need to provide accurate real-time results to be effective. Optical advancements offer potential benefits; however, challenges include cost-effectiveness, device complexity, required training, and tumor heterogeneity.

**Conclusions:**

Optical techniques could accelerate BC diagnosis, reduce costs, and enable alternative treatments. However, overcoming technical and practical challenges is essential for their successful integration.

## 1 Introduction

State-of-the-art research in bladder cancer (BC) diagnosis is being driven by advances in optical technologies, biomarker discovery, non-invasive imaging, and the integration of artificial intelligence into clinical workflows. The goal is to detect BC earlier, more accurately, and with fewer invasive procedures. Our viewpoint is that research and development of optics and photonics will be key to improving outcomes for patients.

Currently, the diagnostic process for BC involves several steps. First, patients presenting with hematuria or abnormalities detected by ultrasound or computed tomography (CT) scan undergo white light cystoscopy (WLC) to identify bladder tumors. If a tumor is found, the patient is scheduled for transurethral resection of the bladder tumor (TURBT). The resected tissue is examined by a pathologist to determine the grade and local stage of the tumor, which is needed for risk stratification to determine the treatment plan.^1^ With a recurrence rate of over 50% after initial treatment,^2^ long-term surveillance is required, leading many patients to repeat this diagnostic process up to every 3 months. Because WLC cannot determine tumor grade and stage, and considering the invasive, time-consuming, and costly nature of the current diagnostic process, there is a growing need for improved diagnostic methods, such as advanced optical technologies. We will outline the challenges associated with current diagnostic methods, the existing application of optics, and the opportunities and drawbacks of optical diagnostic methods in the detection and diagnosis of BC and outline a future roadmap to improve on this.

## 2 Challenges in Bladder Cancer Detection and Diagnosis

### 2.1 Limitations of Cystoscopy

The current role of a WLC in BC diagnosis is to evaluate for tumors or suspicious lesions in the bladder. Unfortunately, WLC is user-dependent and is therefore not accurate for predicting the stage or grade of identified tumors and can have both false positives and false negatives.^3^^–^^5^ Tumor stage is determined by the invasion of BC through the layers of the bladder wall, whereas tumor grade is defined by cytologic features particularly nuclear atypia, which correlates with the degree of cancer aggressiveness. Both stage and grade impact BC management; therefore, accuracy matters for patient care. A recent study in 321 patients with primary BC in the Netherlands showed that WLC has a sensitivity and specificity for predicting muscle invasion of 71.8% and 89.9%, respectively.^3^ In addition, tumor grading is assessed correctly in only ∼55%.^4^ Furthermore, in 17.1% to 52.9% of the TURBTs, the histopathology results show no cancer, indicating a false positive on prior WLC.^5^^,^^6^ Given the combination of cognitive assessment with manual handling of the cystoscope, lesions can easily be missed on WLC, resulting in false negative results.^7^ Results may be poorer than described in the literature when cystoscopies are performed by doctors in training with less experience. Therefore, there exists a need for technologies with better diagnostic characteristics than WLC.

### 2.2 Cystoscopy Limitations Require Histopathologic Specimens

Treatment options for BC diverge radically depending on whether the tumor invades the detrusor muscle of the bladder. Non-muscle invasive bladder cancer (NMIBC) can be treated endoscopically by TURBT followed by possible adjuvant intravesical therapy including bacillus Calmette-Guérin (BCG) and chemotherapy. By contrast, neoadjuvant chemotherapy and radical cystectomy are recommended as first-line treatments for patients with muscle-invasive bladder cancer (MIBC).^1^ The tumor grade, on the other hand, is needed to determine the risk stratification for NMIBC. Other factors for risk stratification include the size and number of tumors, the presence of carcinoma *in situ* (CIS), and depth of tumor invasion.^1^ The risk groups are based on the probability of progression of the NMIBC into MIBC, and recommendations for adjuvant intravesical therapies depend on the risk group the patient is in. Therefore, accurate local tumor staging is paramount to determine the treatment plan.

As WLC cannot provide these critical data points, histopathological analysis from TURBT specimens remains the current gold standard, requiring patients to undergo a second procedure. Despite the reliance on histopathology, inter-observer agreement of BC grading varies widely, with studies reporting agreements ranging from 38% to 89%.^8^ Furthermore, pathologists fail to recognize muscle invasion in 20% to 30% of adequate TURBT specimens.^9^

### 2.3 Costs of the Standard Diagnostic Process

WLC costs range from $172 to $548 in the United States, United Kingdom, and Sweden, as reported by Svatek et al. in 2014, and $485 in 2023 in the Netherlands.^10^^,^^11^ Most patients with BC will require numerous follow-up cystoscopies. The cost of a TURBT ranges from $2318 to $4581, in four European countries and the United States, with a price of $4831 in 2023 in the Netherlands.^10^^,^^11^ There are additional costs, such as productivity losses and travel costs for the patient and family, which have not been taken into account in these prices. Ultimately, the current methods for tumor diagnosis and surveillance via WLCs and TURBTs are costly to health systems and patients. (All prices in euros were converted to US dollars using an exchange rate of 1.05 as of December 2024.)

### 2.4 Available Non-Optical Alternatives

Given the cost and diagnostic characteristics of WLC and TURBT, researchers have evaluated non-optical diagnostic options available for staging and grading BC. Ultrasound, which is low-cost and widely available, is an option. However, limited data exist regarding the use of ultrasound for staging bladder tumors. Micro-ultrasound imaging has demonstrated the capability to describe normal bladder wall layers and identify tumor stage.^12^ Gupta et al. analyzed the accuracy of contrast-enhanced ultrasound (CEUS) in BC, finding sensitivities for staging BC for NMIBC (Ta and T1) and MIBC of 90.0% and 90.7%, respectively. They reported specificities of 75.7% and 92.8% for NMIBC and MIBC, respectively. For grading BC, they found a sensitivity of 78% and a specificity of 85%. Thus, CEUS misclassifies NMIBC as MIBC in ∼10% of patients and high-grade tumors as low-grade in about 15% of cases.^13^ Given the stakes of misclassification, ultrasound in its current form is no alternative to histopathology.

In BC, CT scans are primarily used to identify urothelial cancer in the upper urinary tract and to evaluate for locoregional and distant metastases.^14^ Although CT is able to identify most cases of tumor invasion into perivesical tissue (stage T3b) or tumor growth into surrounding organs or the abdominal wall (stage T4), CT scans lack the soft tissue resolution needed to differentiate NMIBC from MIBC.^14^^,^^15^ Early models have tested machine learning for differentiating between NMIBC and MIBC on CT, but further investigation is needed before clinical utility can be determined.^16^

In recent years, much research has focused on the use of magnetic resonance imaging (MRI) for BC staging. In 2018, the Vesical Imaging Reporting and Data System (VI-RADS), a standardized MRI scoring system for BC, was developed. Studies evaluating MRI with VI-RADS have shown good agreement among radiologists, with sensitivities and specificities ranging from 70.6% to 95% and 44% to 97%, respectively.^17^^–^^19^ Further modifications to VI-RADS are needed to enhance diagnostic validity to be used in lieu of WLC. Similar to CT, MRI cannot provide information regarding tumor grade. In addition, MRI is a costly, time-consuming technique and is not suitable for all patients.

Although imaging cannot currently grade tumors, image-guided biopsy can provide tissue for grading; however, evidence is limited. Chlosta et al. analyzed the use of core biopsy, but only as an alternative for TURBT in patients with extensive, solid tumors suspicious for MIBC to simplify the diagnostic procedure before radical therapy.^20^ Because detrusor muscle in the pathology tissue is needed to identify the tumor stage, a deep biopsy of the bladder wall is needed. Furthermore, due to heterogeneity within bladder tumors, there is a chance of missing tumor invasion or high-grade components of the tumor with core biopsy.^21^^–^^23^

## 3 Current Application of Novel Optical Diagnostic Tools in Bladder Cancer Detection and Diagnosis

Both the European Association of Urology (EAU) and the American Urological Association (AUA) guidelines address the use of enhanced optical techniques in BC diagnosis.^1^^,^^24^ The EAU guidelines recommend the use of photodynamic diagnosis (PDD) or narrow-band imaging (NBI) to enhance tumor visualization during TURBT if these techniques are available. They also mention color space enhancement (IMAGE1S) and confocal laser endomicroscopy (CLE), noting that there is insufficient evidence to support the advantage of IMAGE1S in BC diagnosis and that CLE requires further validation. The AUA guidelines recommend the use of PDD during TURBT to improve detection rates and reduce BC recurrence, if available. They also suggest considering narrow-band imaging (NBI) but do not address other optical techniques. Although NBI was described for BC in 2008 and PDD as early as 1964,^25^^,^^26^ limited data exist on how often these tools are used in clinical practice. With further advances in these technologies and increased clinician familiarity with their use, enhanced optical technologies could play a larger role in BC diagnosis and treatment.

## 4 Opportunities and Challenges of Optical Diagnostic Technologies in Bladder Cancer Diagnosis

### 4.1 Potential Benefits of Optics in Bladder Cancer

We offer the perspective that the implementation of enhanced optical technologies that allow for improved BC diagnosis in real time offers several benefits. First, if an outpatient-usable technique could accurately determine tumor stage and grade in real time, it would accelerate the diagnostic process. Because the TURBT is usually performed several weeks after the cystoscopy and the pathologists require 1 to 2 weeks for the histopathological evaluation, the initiation of definitive treatment could be accelerated by weeks, potentially reducing the risk of disease progression. For example, patients with CIS could start directly with bladder instillations as treatment without waiting for the TURBT and histopathology results, patients with superficial recurrences could be treated with other modalities than the TURBT such as laser fulguration,^27^ and patients with MIBC could be, theoretically, treated directly with curative radical treatment. Second, knowing the stage and grade of the tumor could enable the implementation of other treatment options in selected cases of smaller and noninvasive BC recurrences, such as laser fulguration, active surveillance, or chemoablation.^27^^–^^29^ Third, decreased reliance on TURBTs, especially in cases of questionable lesions seen on WLC, would result in fewer surgery-associated complications for patients, such as bleeding, perforation, infection, and complications of the anesthetics. In addition, the reduction in TURBT procedures and associated complications could lead to a decrease in the overall costs of BC diagnosis and management, including patient and productivity costs, once the expenses of the technique itself are offset. Furthermore, improved BC detection is known to decrease the recurrence of BC.^30^

### 4.2 Challenges for Optical Technologies in Bladder Cancer

We believe that the future development of optical techniques for diagnosing BC faces several challenges. To be of the highest value in BC diagnosis, the technique should be suitable for outpatient clinic use. Consequently, compatibility with a flexible cystoscope, which typically has a diameter of ∼2.2  mm,^31^^,^^32^ is required, and the technique must deliver rapid real-time results. Furthermore, the balloon shape of the bladder requires techniques to be flexible to access all locations within the bladder. Any technology needs to be suitable for use in an aqueous environment and in contact with urine. Because the bladder collapses when empty, the bladder needs to be distended with either urine or an irrigant to enable visualization of the bladder wall. Heterogeneity is also a challenge for optics in BC diagnosis.^21^^–^^23^ To effectively address the heterogeneity of BC, optical techniques may need to possess either a wide field of view, the ability to maneuver easily within the bladder, or a combination of both, to ensure comprehensive tumor assessment. Achieving these requirements may be a challenge.

Moreover, before a new technique can be implemented, it must prove cost-effectiveness.^33^ Even if a technique demonstrates cost-effectiveness, its initial purchase costs must be feasible for hospitals with diverse budgets, given that BC diagnosis is a routine procedure for most urologists. The substantial development costs associated with optical techniques present a significant barrier. Another critical challenge is the learning curve associated with adopting a new optical technique. Procedures in outpatient clinics are performed not only by experienced urologists but also by urologists in training and those who frequently change institutions with different equipment. Therefore, a short learning curve is essential to maintain diagnostic accuracy and ensure the widespread use of the optical technique. Ensuring interpretability poses yet another challenge. The interpretability of results by urologists, who will utilize the technique, is crucial. This necessitates close collaboration between technology developers and urologists throughout the technique’s development process, which may prove challenging, especially for new start-ups in the field. Researchers and product engineers looking to work in this field should keep these challenges in mind when developing new technologies.

## 5 Perspective on Optical Techniques with the Potential to Diagnose Bladder Cancer

**Fig. 1:**
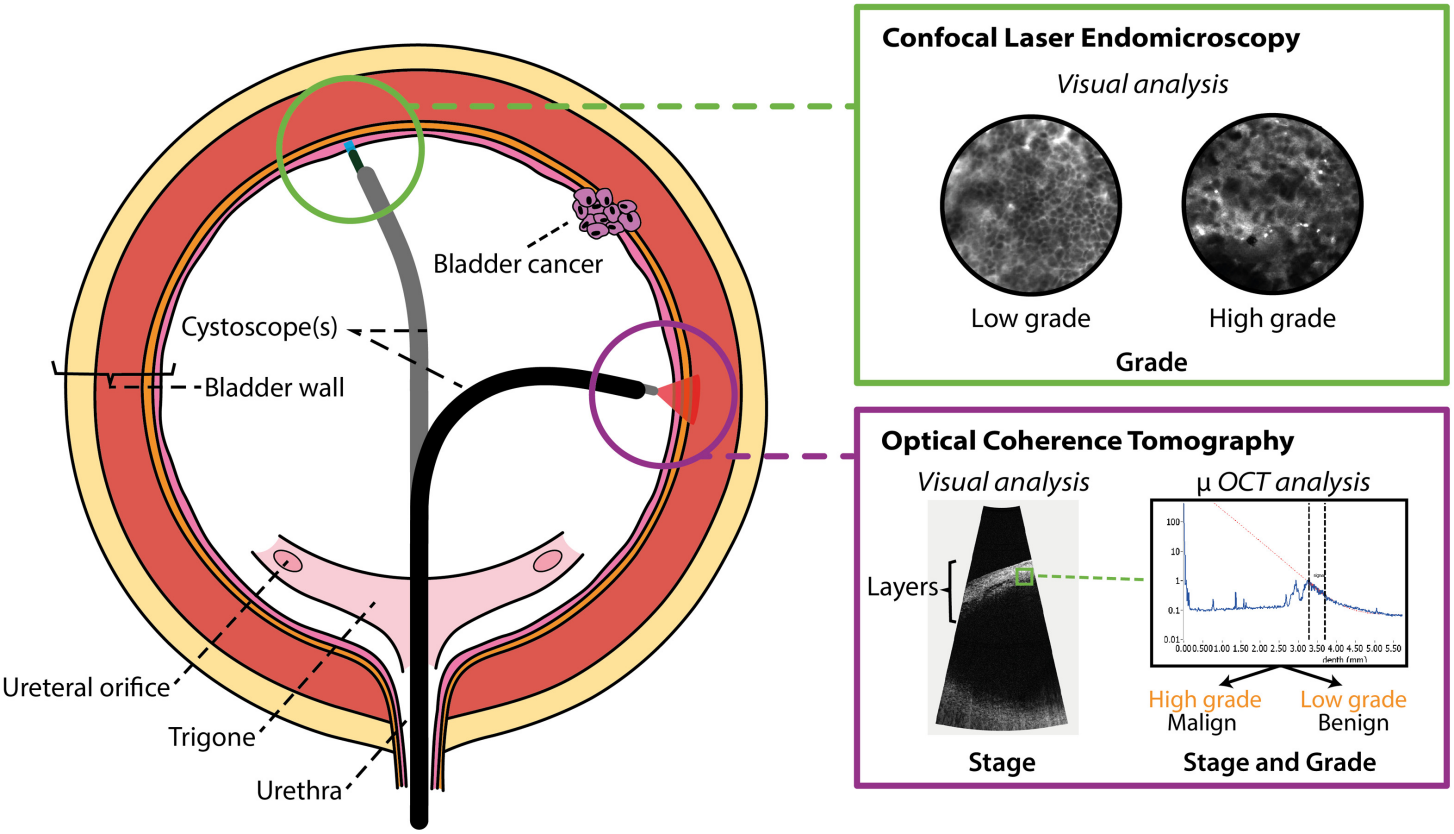
Promising optical techniques for bladder cancer diagnosis. CLE and OCT are two promising optical techniques for improving BC diagnosis. Both methods have the potential to access the bladder endoscopically using a flexible cystoscope, as illustrated. OCT may aid in staging BC by imaging the bladder wall layers and evaluating tumor invasion, with the attenuation coefficient (μOCT) potentially helping to distinguish tissue characteristics. In addition, μ OCT may aid in grading BC by detecting variations in attenuation among different cancer grades. CLE, on the other hand, may facilitate cancer grading by enabling the visualization of cellular structures and morphological differences. OCT = optical coherence tomography, μ = attenuation coefficient.

In our view, which is mostly shared by the authors of the EAU guidelines on NMIBC, several optical techniques hold real potential to aid in the diagnosis of BC.^1^ One promising technique is CLE, which captures high-resolution images of tissue at the cellular level by reflecting laser light. In the case of BC, fluorescence is used to enhance contrast and highlight specific tissue components more clearly.^34^ Thereby, it offers the ability to grade BC in a less invasive manner compared with TURBT (see Fig. 1).

Studies have reported sensitivities and specificities of 22% to 94.5% and 66.7% to 96% for grading BC, using a CLE catheter compatible with a rigid cystoscope.^35^ CLE provides real-time imaging, and when combined with artificial intelligence (AI), it could enable real-time interpretable grading, eliminating the need for the traditional histopathological analysis that takes 1 to 2 weeks. Recent research by Lucas et al. has demonstrated that CLE, in combination with AI, can differentiate between low-grade and high-grade NMIBC.^36^ However, with an imaging depth of only 70  μm, CLE cannot visualize deeper layers of the bladder wall, which is essential for accurate tumor staging.^34^ In addition, CLE in BC diagnosis requires the use of fluorescent contrast agents, increasing the risks for patients and increasing its overall cost. Despite these limitations, we believe CLE could play a valuable role in BC diagnosis when integrated with a technique capable of noninvasive staging.

A technique that, to our knowledge, holds potential for both grading and staging BC is optical coherence tomography (OCT). OCT offers an imaging depth of ∼2  mm in tissue, and because the detrusor muscle begins at a depth of around 0.6 to 3.5 mm, it may be capable of accurately staging BC.^37^^,^^38^ Similar to CLE, OCT provides real-time imaging, significantly accelerating the diagnostic process. A key advantage of OCT is that its attenuation coefficient can aid in tumor identification and may even assist in determining tumor grade (see Fig. 1). Previous studies have shown that OCT can distinguish between high-grade and low-grade urothelial carcinoma in the upper urinary tract based on the attenuation coefficient.^39^^,^^40^ When combined with machine learning or AI, OCT could potentially enable highly accurate real-time interpretation of tumor stage and grade. However, because OCT typically operates as a side-viewing technology, which is not ideal for a balloon-shaped organ such as the bladder, a forward-looking catheter is necessary. Such a forward-looking OCT system has been developed and successfully tested *ex vivo*, recently highlighting its potential as a diagnostic tool for BC.^41^

Other optical techniques, such as Raman spectroscopy and nonlinear optics (NLO), have been explored or proposed as potential diagnostic tools for BC.^35^ However, these methods require significant further development before they can be considered viable options for BC diagnosis. Raman spectroscopy is not yet a viable diagnostic tool for urologists, due to the lack of interpretability and the fact that it is not yet robust enough for use in measuring in the bladder. Furthermore, the optics of NLO are difficult to process into a probe-based optical device and currently do not enable direct interpretation of the data.

Ultimately, the technological solution that offers sufficient contrast and resolution to find, stage, and grade BC accurately in real time will likely require a combination of optical and non-optical techniques. We anticipate that WLC will remain the primary technology for comprehensive bladder surveillance. However, by integrating WLC with advanced image reconstruction algorithms, it may become possible to generate a full bladder atlas, where every suspected lesion is delineated using AI-assisted segmentation. Recent work by Groenhuis et al. has demonstrated the feasibility of full bladder map reconstruction using computer vision and AI-assisted detection of BC in WLC, as described by Chang et al., showing potential for reducing missed lesions.^42^^–^^45^ To complement this approach, an optical imaging technique—most likely OCT—also combined with AI could be used to determine the stage and grade of identified tumor(s). To accurately evaluate the extent of tumor invasion, particularly beyond the musculus detrusor, we believe supplementary technologies are necessary, as OCT and CLE are limited by relatively shallow imaging depths. In this regard, low-field MRI is the most promising solution in our opinion. Low-field MRI is both faster and more cost-effective than conventional MRI,^46^ and ongoing research into BC staging using MRI further supports its potential role.^47^^,^^48^ Considering these advancements, we believe that integrating enhanced optical technologies (e.g., CLE or OCT), external imaging modalities (e.g., MRI), and AI could provide a comprehensive real-time solution for improving BC diagnosis and surveillance. However, forward-looking OCT is not yet compatible with a flexible cystoscope, whereas CLE, although compatible, lacks sufficient resolution to maintain its accuracy, and MRI currently lacks the necessary precision. In addition, the cost-effectiveness of these potential techniques, whether used independently or as part of a combined solution, remains to be determined in the future.

## 6 Conclusions

BC diagnosis and surveillance in its current paradigm is costly, time-consuming, and limited by reliance on TURBT for histologic analysis. There are currently no available non-optical alternatives that can provide the necessary information, such as tumor grade and stage, for making treatment decisions. Developing enhanced optical methods for BC diagnosis presents several challenges. These include challenges inherent to accessing and evaluating the bladder, ensuring affordability for hospitals, addressing the learning curve, ensuring the interpretability of results by urologists, and meeting the histopathological requirements necessary for a comprehensive diagnosis. Nonetheless, future progress in enhanced optical techniques such as CLE and OCT, possibly in combination with AI, holds the potential to improve the diagnostic process in BC and subsequently improve patient outcomes.

## Data Availability

Data sharing is not applicable to this article, as no new data were created or analyzed.
